# In Silico Characterization of Conserved Epitopes in Alphavirus E2 Proteins: A Promising Approach for Pan-vaccine Design

**DOI:** 10.1021/acsomega.5c07474

**Published:** 2025-11-26

**Authors:** Ubiratan da Silva Batista, Ana Clara Gomes de Souza, Breno de Mello Silva, Ricardo Lemes Gonçalves

**Affiliations:** Laboratory of Biology and Technology of Microorganisms (LBTM), Department of Biological Sciences, 28115 Federal University of Ouro Preto (UFOP), Ouro Preto, MG 35400-000, Brazil

## Abstract

Alphaviruses infect a wide range of hosts, including humans and domestic animals, and they represent an increasing public health concern. Among them, arthritogenic Chikungunya virus (CHIKV) and encephalitogenic Eastern equine encephalitis virus (EEEV) stand out for their epidemic potential and clinical severity. Developing effective and licensed vaccine models against these viruses remains a significant challenge. Rational epitope design, supported by immunoinformatics, offers a promising route for next-generation effective vaccine development. In this study, we utilized the POA pipeline to assist in the selection and prioritization of predicted epitopes from the E2 glycoproteins of CHIKV and EEEV. A total of 39 conserved linear epitopes were selected, comprising 8 B-cell, 2 T-cell, and 29 Th-cell epitopes. These epitopes were characterized for allergenicity, toxicity, and physicochemical properties, including polarity and hydrogen-bonding potential. Structural mapping onto the quasi-3-fold (q3) symmetry unit enabled assessment of their solvent accessibility and spatial organization in the native quaternary context. Our result provides a basis for the rational design of a multiepitope vaccine targeting conserved antigenic regions in alphaviruses with high translational relevance. This integrative approach aligns with the One Health perspective, highlighting its potential for developing biotechnological solutions that address human, animal, and environmental health. The POA pipeline is available on GitHub (https://github.com/UbiratanBatista/POA_Project).

## Graphical abstract

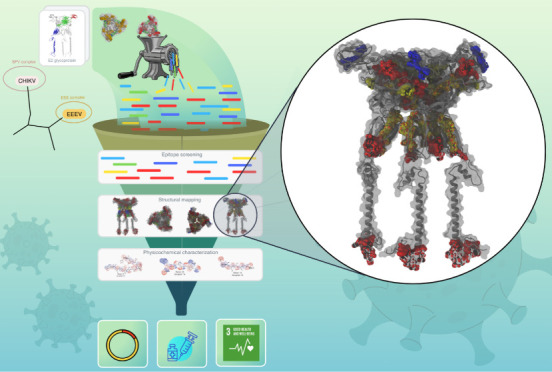

## Introduction

Alphaviruses are emerging arboviruses of major public health concern, responsible for outbreaks that cause severe disease in both humans and animals worldwide.^1^ The genus Alphavirus (family *Togaviridae*) comprises ∼30 enveloped, single-stranded, positive-sense RNA viruses, which are broadly classified into arthritogenic and encephalitogenic groups based on clinical manifestations and epidemiological patterns. Chikungunya virus (CHIKV), an arthritogenic alphavirus, causes febrile illness, skin rashes, and debilitating polyarthritis.^2^ In contrast, eastern equine encephalitis virus (EEEV), a representative encephalitic alphavirus, is associated with acute and often fatal neurological disease.^3^ Both viruses exemplify the epidemic potential of alphaviruses and their capacity to cause large-scale outbreaks.^4^

Despite their clinical relevance,^5,6^ effective vaccines and antivirals for human use remain limited. To date, two vaccines against CHIKV infection have received regulatory approval,^7,8^ while additional candidates are progressing through advanced clinical evaluation. In contrast, vaccine development for other alphaviruses has lagged significantly.^9,10^ For EEEV, for example, available licensed formulations are restricted to veterinary use in equines and typically elicit moderate immunogenicity,^10^ requiring periodic booster doses to maintain protective titers.^6,9^ These gaps underscore the need for rational vaccine strategies designed to target both arthritogenic and encephalitic alphaviruses.

The E2 structural glycoprotein plays a central role in alphavirus infectivity and immunogenicity, mediating receptor binding and entry into host cells. It is the primary target of neutralizing antibodies and thus represents a critical antigen for vaccine design.^11^ Several antibody-binding epitopes have been identified within the A and B ectodomains of E2,^12^ and cross-neutralizing monoclonal antibodies have been reported for certain arthritogenic alphaviruses.^13^ However, equivalent antibodies remain elusive for encephalitic alphaviruses,^9^ suggesting that identifying conserved antigenic regions across phylogenetically distinct alphaviruses may represent a promising avenue for the development of pan-vaccines, diagnostics, and immunotherapies. An understanding of the architecture and molecular properties of these conserved epitopes may support an integrated “One Health” approach to protect both human and animal populations.^14^

Structural analyses have revealed that alphavirus spike particles share a conserved architecture, composed of trimers of E1–E2 heterodimers arranged along the icosahedral axes of symmetry.^15^ High-resolution cryoelectron microscopy (cryo-EM) of Mayaro virus (MAYV) particles further resolved the quasi-3-fold (q3) symmetry unit of the viral spike, providing a biologically relevant framework for studying epitope accessibility.^16^ These structural insights emphasize the importance of considering quaternary arrangements when defining immunogenic regions since solvent-exposed epitopes on the native viral particle may differ significantly from those inferred from isolated proteins.^17–19^

*In silico* linear epitope prediction has accelerated the identification of B- and T-cell targets, but conventional approaches often lack integration of physicochemical characterization and quaternary structural context during epitope screening.^20,21^ Antigen–antibody interactions are determined not only by sequence conservation and surface exposure but also by intramolecular interaction networks and the local structural environment. Cryo-EM studies of CHIKV in complex with Fab fragments of mAb CHK-263 (PDB ID: 7CW2) revealed that neutralizing antibodies preferentially target epitopes located near solvent-exposed vertices, where dense polar interactions between viral residues and hypervariable loops of the antibody complementarity-determining regions (CDRs) stabilize binding^22,23^ and prevent viral entry or membrane fusion.^24,25^ These findings underscore the importance of coupling epitope prediction with structural and physicochemical validation, as discrepancies between predicted and experimentally validated epitopes can significantly compromise translational reliability and posing a major bottleneck in the rational design of vaccine candidates.^4,20^

To address these challenges, we developed an integrated strategy that leverages the biologically relevant q3 unit of the alphavirus spike to refine epitope selection. Using multiple immunoinformatics tools, a novel set of linear B- and T-cell epitopes targeting the E2 glycoproteins of CHIKV and EEEV was predicted and subsequently screened through the semiautomated pipeline for optimization of antigens (POA) (Figure 1). The selected epitopes were systematically mapped onto E2 structural models, evaluated for solvent accessibility, and characterized according to their hydrogen-bonding potential. This integrative analysis generated a refined antigenic landscape of the E2 glycoprotein and identified high-confidence candidates for the development of cross-protective alphavirus vaccines. By coupling cryo-EM structural data with multiplatform immunoinformatics, this approach facilitates the identification of conserved, solvent-exposed, and physicochemically favorable epitopes—principles that can be broadly applied to other Alphavirus species.^26,27^

**Figure 1:**
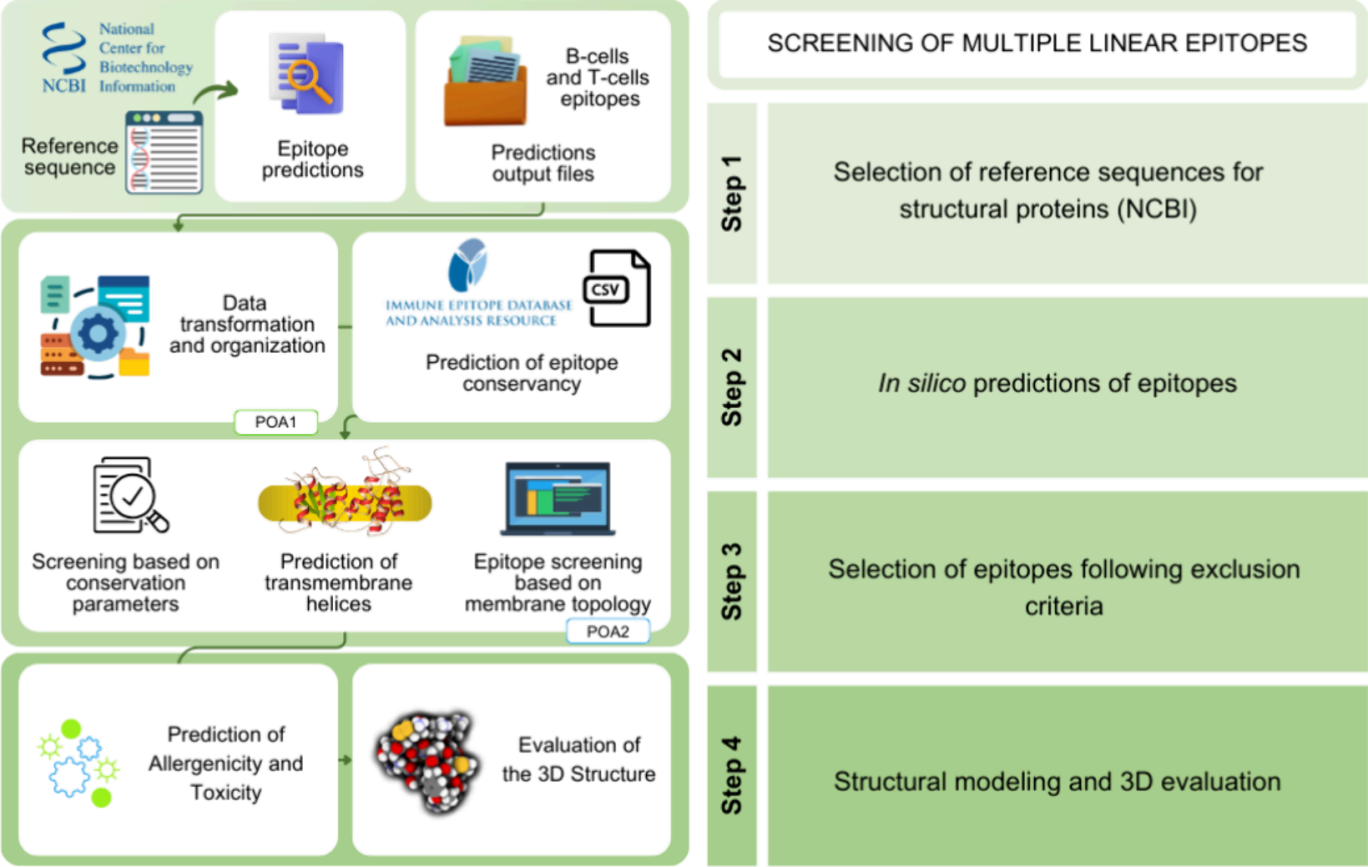
Schematic overview of the pipeline for optimization of antigens (POA) used to select optimal B- and T-cell epitopes from structural proteins for the design of a multiepitope vaccine targeting a consensus Alphavirus antigen. Epitopes are predicted from protein sequences using web-based immunoinformatics tools and then ranked and filtered based on conservation across viral species, surface exposure, and predicted toxicity or allergenicity. Finally, selected epitopes are structurally mapped to determine their spatial distribution and accessibility within the viral particle.

## Materials and Methods

### Retrieval of Alphavirus Proteins

The E2 glycoprotein sequences were derived from the structural polyprotein sequences of arthritogenic CHIKV (accession at NCBI: NP_690589.2) and encephalitogenic EEEV (accession at NCBI: NP_632022.1) viruses. The structural polyproteins were translated from the reference genomes of these species, as reported by Khan et al.^28^ Protein sequence data were retrieved from the NCBI Protein database on July 14, 2021 (Box S1). The sequences underwent assessment for completeness using the Filter Protein tool in the Sequence Manipulation Suite^29^ and were stored in fasta files in a dedicated database.

### Epitope Prediction

For the prediction of epitopes within the E2 proteins, we employed the following computational tools: Bepipred 2.0 ^30^ (https://services.healthtech.dtu.dk/service.php?BepiPred-2.0) and Predicted Antigenic Peptides (PAP)^31^ (http://imed.med.ucm.es/Tools/antigenic.pl) for B-cell epitopes, NetCTL 1.2 ^32^ (https://services.healthtech.dtu.dk/service.php?NetCTL-1.2) for cytotoxic T-cell (Tc) epitopes, and MHCII Binding Predictions^33^ (http://tools.iedb.org/mhcii/) for T helper (Th)-cell epitopes. The results generated by these prediction methods are publicly accessible at 10.5281/zenodo.14968128.

The B-cell epitope predictions were conducted using the default settings for the methods. The results obtained from Bepipred predictions were downloaded in the JSON file format. Bepipred 2.0 employs a sequence-based model, utilizing a random forest regression algorithm trained on annotated epitopes from crystallographic structures of antibody–antigen complexes.^30^ In the case of PAP, the model calculates the average probability of the epitope antigenic potential based on the occurrence of conserved amino acid residues in experimentally determined epitopes.^34^ The table containing the results of the PAP method predictions was saved in a text file format.

Tc-cell epitope predictions were conducted using the standard method of the NetCTL tool, and the resulting epitope table was downloaded as an HTML file. The NetCTL model employs neural network algorithms to predict peptide binding to MHC class I, proteasomal cleavage prediction, and a weight matrix for predicting the efficiency of transport by the TAP transporter.^32^

Finally, the Th-cell epitope prediction was performed for the human HLA-DR locus using the reference allele set provided by the MHCII Binding Predictions tool.^32^ The method used was IEDB recommended 2.22. With the remaining parameters set to their default values, the results table containing values from various peptide-MHC class II binding predictions was saved as an HTML file.

### Screening the Best Candidates from the Prediction Results

The results from the epitope prediction methods were processed through the first module of the semiautomatic pipeline for optimization of antigens, POA1 (Figure 1). This module is responsible for screening and organizing the most promising epitopes based on the prediction scores assigned by each method. The algorithm structures the prediction outputs into a relational data model, returning a FASTA file that contains detailed information for each selected epitope, including the organism name, protein of origin, prediction method, sequence position, and amino acid sequence. This organization facilitates analyses and ensures reproducibility across subsequent stages of the pipeline.

For the POA1 analysis, some parameters were adjusted to improve and refine the resulting epitope data set. B-cell epitopes were filtered by sequence length, with a minimum cutoff of six amino acid residues. For Th-cell epitopes, candidates were prioritized according to binding affinity, with an IC_50_ threshold of ≤25 nM, corresponding to the top half of epitopes classified as strong binders (IC_50_ < 50 nM) to the human HLA-DR allele.^35,36^ These criteria ensured that only epitopes with higher predicted immunogenic relevance were retained for further evaluation. Redundant Th-cell epitopes predicted across multiple HLA alleles were removed for further analysis (Table S1).

### Epitope Conservancy Analysis and Transmembrane Helix Prediction

The epitopes selected by POA1, along with the structural polyprotein of both viruses, were subjected to conservation analysis using the IEDB Epitope Conservancy Analysis tool^37^ (http://tools.iedb.org/conservancy). To avoid redundancy, epitopes derived from the E2 protein of one virus were compared to the polyprotein sequence of the other. Sequence identity thresholds were incrementally evaluated (50–90%), and a minimum cutoff of 60% identity was adopted, as higher conservation values (70–71%) were observed only for two epitopes. Thus, linear epitopes were considered conserved when they met or exceeded the 60% identity threshold in the IEDB.

The resulting CSV files from the conservancy analysis were processed through POA2 (Figure 1), the second module of the POA pipeline. POA2 analyzes the conservancy data for each peptide, selecting epitopes based on user-defined thresholds and ranking them according to similarity or exclusivity. For this study, the 60% identity cutoff was maintained to ensure consistency with IEDB analysis. Within the POA2 execution, the selected epitopes were also characterized for membrane topology using the integrated pyTMHMM^38^ module (https://github.com/bosborne/pyTMHMM), a tool based on hidden Markov models to predict transmembrane helices. The epitopes characterized during the analysis are summarized in Table S2.

### Toxicity and Allergenicity Analysis

The previously selected epitopes underwent toxicity and allergenicity analysis. For these predictions, we utilized the web prediction tools Toxinpred (https://webs.iiitd.edu.in/raghava/toxinpred/index.html) and Allercatpro (https://allercatpro.bii.a-star.edu.sg/), respectively.^39,40^ The results did not indicate a toxic or allergenic potential for any predicted peptides.

### Sequence Similarity and Conserved Antigenic Motifs in Protein E2

The regions of sequence similarity between the E2 proteins of CHIKV and EEEV were assessed using pairwise alignment with the EMBOSS^41^ Needle web tool (https://www.ebi.ac.uk/Tools/psa/emboss_needle/), configured to output results in a “pair” format. The analysis revealed identity and similarity values of 41.3 and 56.2%, respectively. Conserved antigenic sites were identified through multiple sequence alignment of the E2 protein sequences and predicted epitopes, performed using the ClustalW algorithm^42^ implemented in MEGA-X software.^43^ The results were visualized using the ESPript tool^44^ (https://espript.ibcp.fr/ESPript/ESPript/).

### Identification and Characterization of the q3 Structural Unit

To establish a reliable structural framework for epitope mapping, we first analyzed the high-resolution cryo-EM three-dimensional structure of the MAYV particle (PDB ID: 7KO8). Structural visualization was performed using BIOVIA Discovery Studio.^45^ A representative quasi-3-fold (q3) symmetry unit, composed of E1–E2 heterodimers, was extracted. This unit provides a complete polyprotein chain and reflects the biologically relevant better arrangement of spikes on the viral surface, thereby serving as a reference model for assessing epitope exposure.^16^ The structural insights obtained from MAYV were then applied to guide the mapping of epitopes on crystallographic models of CHIKV and EEEV E2 glycoproteins.

### 3D Characterization of Epitopes and Accessible Solvent Area (SASA Å^2^)

The crystal structures of the E2 glycoproteins from CHIKV (PDB ID: 6NK7, 4.99 Å)^41^ and EEEV (PDB ID: 6MX4, 4.40 Å)^42^ were retrieved from the RCSB Protein Data Bank (https://www.rcsb.org/).^43^ Structural evaluation and validation were performed using MolProbity (Ramachandran plots and statistics, Clashscore, and MolProbity score),^44^ ProSA-web (*Z*-scores),^45^ and QMEAN (Qualitative Model Energy Analysis) (10.1093/bioinformatics/btq662) (https://swissmodel.expasy.org/qmean). For the QMEAN evaluation, all ligand molecules were removed from the original PDB files. The CHIKV E2 structure exhibited a Clashscore of 16.31 (97th percentile), a MolProbity score of 2.30 (99th percentile), a *Z*-score of −5.29, with 99.9% of residues in favorable regions of the Ramachandran plot, and a QMEAN score of −3.45 (Figure S1). The EEEV E2 crystal showed a Clashscore of 7.5 (97th percentile), a MolProbity score of 2.14 (100th percentile), a *Z*-score of −5.27, with 99.5% of residues in favorable regions, and a QMEAN score of −4.66 (Figure S2).

Three-dimensional visualization of conserved epitopes was performed using BIOVIA Discovery Studio 2019, and the total surface area of each epitope was determined using the get_area function in PyMOL (https://pymol.org/2/). Figure rendering was performed using VMD (University of Illinois).^46^ The solvent-accessible surface area (SASA), buried surface area (BSA), and hydrogen-bonding interactions within the q3 heterodimer context were calculated using the PDBePISA web server (https://www.ebi.ac.uk/pdbe/pisa/),^47^ followed by manual inspection. Because molecular interface analysis requires careful interpretation to accurately describe intermolecular interactions, the PDBePISA results were further cross-validated using COCOMAPS 2.0 (https://aocdweb.com/BioTools/cocomaps2/), a web tool that employs intermolecular contact maps to identify, analyze, and compare protein–protein interface interactions^48^ (Table S3).

### Mapping Hydrogen Bond Donors and Acceptors on Viral Epitope Surfaces

This step was conducted using structural data from the q3 unit of the CHIKV E protein homotrimer, obtained from the Protein Data Bank (PDB) with accession codes 6NK7 ^49^ for CHIKV and 6MX4 ^50^ for EEEV. Structural modeling and visualization of the molecular surfaces for each epitope were performed using BIOVIA Discovery Studio software, with the standard configuration tools,^51^ to determine all features, preserving the original conformation of the q3 unit. For each predicted epitope, the corresponding regions in the three-dimensional structures were isolated, and chemical groups capable of acting as hydrogen bond donors (e.g., −NH AND −OH) or acceptors (e.g., oxygen or nitrogen with lone electron pairs) were quantified. The analyzed regions were selected based on previously identified epitopes, prioritizing those with high binding affinity and conservation. The BIOVIA Discovery Studio^51^ Tool was already used to analyze the features of the interface of interactions of a neutralizing mAbs bound to E2 CHIKV VLPs (PDB ID: 8dww)

## Results

### Selection of Conserved Antigenic Epitopes

Putative epitopes for B cells, cytotoxic T (Tc) cells, and helper T (Th) cells were predicted from the E2 glycoproteins of CHIKV (NCBI accession: NP_690589.2) and EEEV (NCBI accession: NP_632022.1). The raw predictions were first processed using the POA1 module, resulting in the selection of 30 B-cell, 11 Tc-cell, and 88 Th-cell epitopes for CHIKV and 26 B-cell, 16 Tc-cell, and 123 Th-cell epitopes for EEEV (Table 1). After POA1 processing, the largest reduction occurred among the Th-cell epitope group due to prioritization of peptides with high predicted MHCII binding affinity (IC_5__0_ ≤ 25 nM).

**Table 1:** Selection of Epitopes by the First Step of the Epitope Optimization Pipeline (POA1)

	Chikungunya virus				Eastern equine encephalitis virus			
prediction method^a^	predicted epitopes	epitopes selected by POA1	average length (aa)	epitopes excluded (%)^b^	predicted epitopes	epitopes selected by POA1	average length (aa)	epitopes excluded (%)^b^
Bepipred	14	11	12.71 ± 9.74	∼21.5	14	13	12.07 ± 8.66	∼7.0
PAP	19	19	14.21 ± 7.29		13	13	22.38 ± 16.71	
NetCTL	11	11	9		16	16	9	
MHCIIBP	1331	88	15	∼93.5	1614	123	15	∼92.5

a We screened epitopes predicted for B cells (Bepipred and PAP), Tc cells (NetCTL), and Th cells (MHCIIBP).

b There was no difference between selected and predicted PAP and NetCTL epitopes.

Epitope conservancy analysis (≥60% identity threshold), followed by POA2 filtering, identified 21 conserved epitopes in CHIKV and 19 in EEEV. In CHIKV, these comprised seven B-cell and 14 Th-cell epitopes, while in EEEV, they included two B-cell, two Tc-cell, and 15 Th-cell epitopes.

Membrane topology prediction revealed that four CHIKV and six EEEV epitopes were located in the internal membrane regions (Figure 2). Conserved B-cell epitopes predicted in internal sites were excluded from downstream structural and accessibility analyses since only surface-exposed epitopes are accessible to neutralizing antibodies. No conserved predicted epitopes were located within transmembrane helices of the E2 proteins.

**Figure 2:**
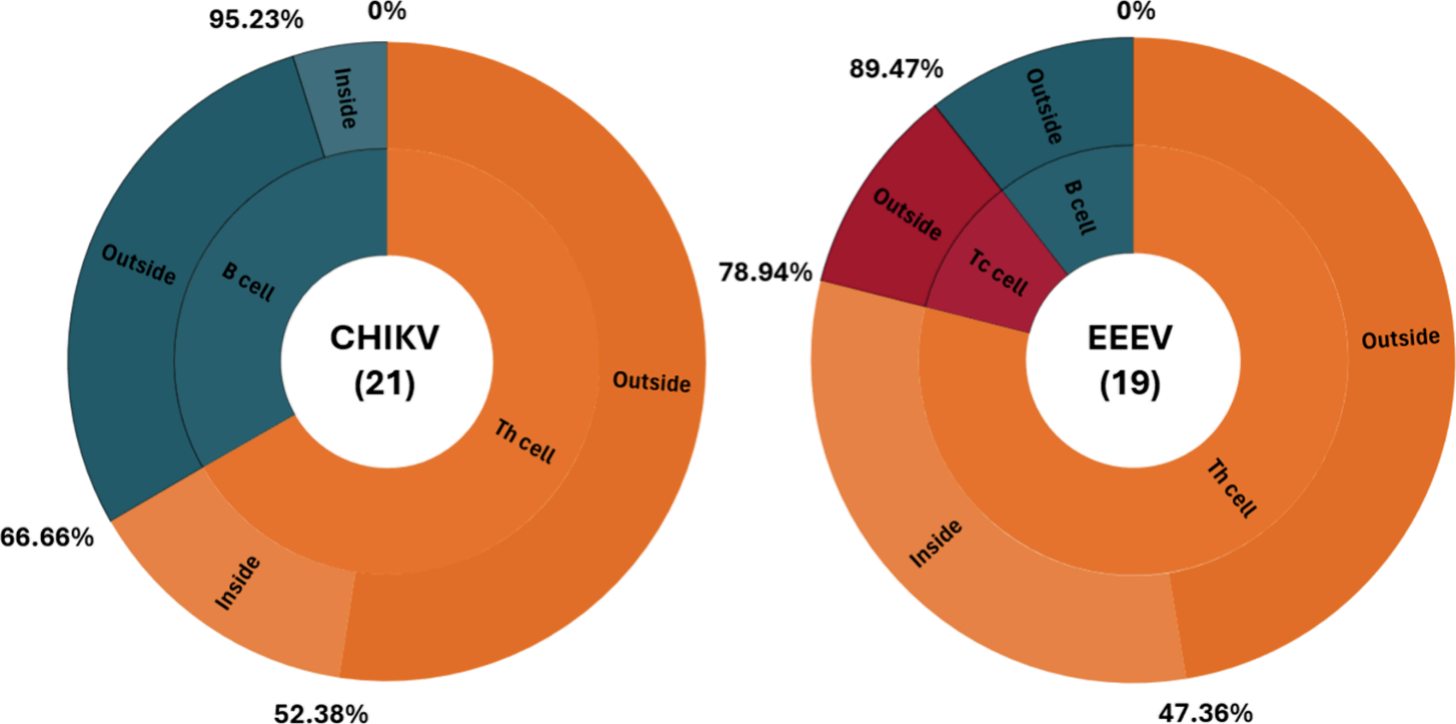
Distribution of epitopes selected by POA in the E2 protein of CHIKV and EEEV. Sunburst charts illustrate the proportion of predicted epitopes for B cells, helper T cells (Th), and cytotoxic T cells (Tc) in CHIKV (21 epitopes) and EEEV (19 epitopes). The inner ring represents the classification of epitopes based on the immune cell type for which they were predicted, while the outer ring indicates their structural topology within the E2 protein (surface-exposed, “Outside”; buried, “Inside”). Most B-cell epitopes are located on the surface. No Tc epitopes were predicted for CHIKV, whereas a small proportion was identified in EEEV.

### B-Cell Epitopes

Seven conserved B-cell epitopes were identified in CHIKV and two in EEEV E2 glycoproteins (Table S2). In CHIKV, epitopes were distributed across all three ectodomains, with four of seven located in ectodomain A. Only one conserved epitope was predicted in ectodomain B (residues 207–212, NEGLIT). Two epitopes, CHIKV 237–244 (YNSPLVPR) and EEEV 142–152 (PEHGVELPCNR), were mapped to the β-ribbon adjacent to ectodomain B. Although this region seems structurally flexible, it is stabilized within E2 heterodimers by interactions with domain A and the E1/E3 proteins.^52^

Toward the C-terminal portion of the protein, a near-membrane-exposed epitope, CHIKV 282–297 (QVIMLLYPDHPTLLSY), was identified in ectodomain C, overlapping with a conserved immunogenic region (residues 284–305). This sequence was predicted to be both a B- and T-cell epitope, sharing 62.5% sequence identity between CHIKV and EEEV. Despite the partial shielding of ectodomain C due to its proximity to the viral membrane, one additional epitope, EEEV 323–332 (LEYTWGNHPP), was also identified in this region. Topology predictions suggested an exposed orientation, supporting its accessibility to the immune system.

In contrast, the CHIKV 404–419 (PGATVPFLLSLICCIR), located within the cytoplasmic tail, was predicted to adopt an internal-membrane-associated topology and was excluded from further analyses.

### T-Cell Epitopes

A total of 27 Tc-cell epitopes were predicted across E2 proteins, of which only two were conserved: EEEV 145–153 (GVELPCNRY) and EEEV 317–325 (TVTGEGLEY), each sharing 66.7% identity with CHIKV. No conserved Tc-cell epitopes were identified in CHIKV.

For Th cells, initial predictions yielded 88 candidates in CHIKV and 123 candidates in EEEV. After the POA filter was applied (IC_5__0_ ≤ 25 nM) and redundant HLA assignments were eliminated, 14 and 15 epitopes, respectively, were retained. Among these, five CHIKV and 10 EEEV epitopes were predicted to bind multiple HLA-DR alleles (≥2) (Table S1), suggesting potential for broad immune coverage.^53^

Two Th-cell epitopes in CHIKV and three in EEEV were mapped to the N-terminal region of domain A, the largest domain of E2. In EEEV, an additional four conserved Th-cell epitopes were predicted within ectodomain B (residues 174–191). No homologous epitopes were found in CHIKV for this region, despite ≥60% sequence conservation (Table S2).

Notably, conserved Th-cell epitopes clustered in two other regions (residues 280–305 and 397–413), both of which showed ∼66.7% conservation between the two viruses. The former corresponded to an external topology in ectodomain C, while the latter localized to a membrane-associated internal region. One epitope in the ectodomain C of CHIKV, 282–297 (QVIMLLYPDHPTLLSY), was predicted to be recognized by both B and Th cells, highlighting a potential immunodominant cluster capable of coordinating humoral and cellular responses.

### Conserved Antigenic Motifs

Pairwise alignment of CHIKV and EEEV E2 proteins revealed overall sequence identity and similarity of 41.3 and 56.2%, respectively, consistent with their evolutionary divergence.^54^ Despite this divergence, three conserved antigenic motifs were identified: (i) residues 6–23 at the N-terminus, (ii) residues 284–296 within ectodomain C, and (iii) residues 397–419 in the C-terminal cytoplasmic tail (Figure 3).

**Figure 3:**
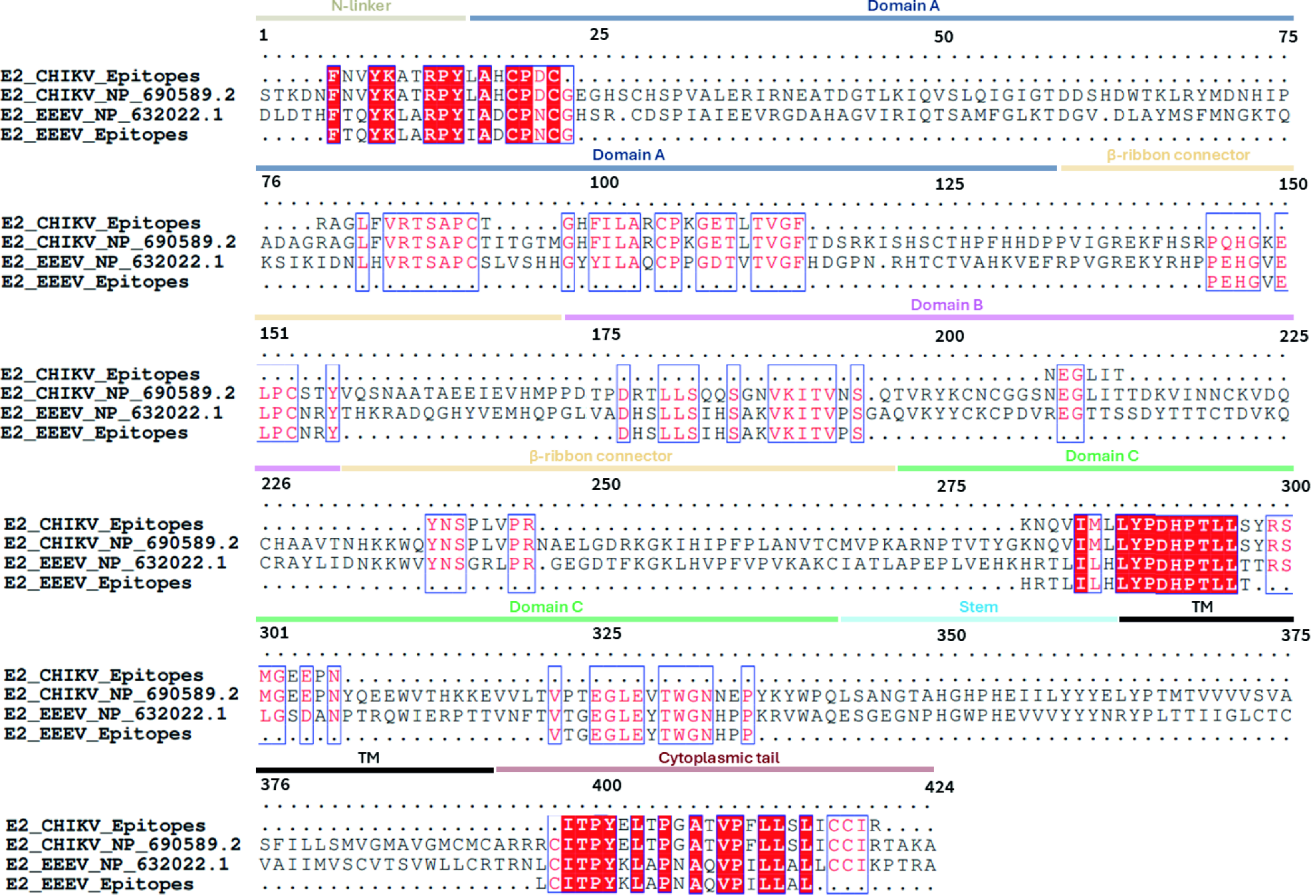
Multiple sequence alignment of the E2 protein from CHIKV and EEEV, highlighting selected epitopes by POA. The alignment compares the E2 protein sequences of CHIKV (GenBank NP_690589.2) and EEEV (GenBank NP_632022.1) against the identified epitopes. Conserved antigenic motifs between protein sequences are highlighted in red.

Most epitopes within these motifs were predicted as Th-cell epitopes, highlighting their potential relevance for CD4^+^ T-cell-mediated responses. Only one B-cell epitope, CHIKV 282–297 (QVIMLLYPDHPTLLSY), overlapped with the ectodomain C motif, presenting an external membrane-associated topology. No conserved Tc-cell epitopes were mapped within these motifs.

The structural and functional roles of these regions may explain their evolutionary conservation. The N-terminal motif includes the N-linker (residues 6–15) and β-hairpin (residues 16–23) of ectodomain A, which stabilize interactions with the E3 protein and contribute to the Ig-like scaffold.^52^ The ectodomain C motif corresponds to critical interdimer contacts required for viral spike trimerization.^15^ Finally, the C-terminal motif encompasses conserved sequences interacting with a hydrophobic pocket of the capsid protein, a step essential for virion assembly and stability.^55^

The identification of epitopes in both conserved motifs and structurally constrained regions provides a valuable foundation for rational vaccine design targeting multiple alphavirus species.^56^ Together, the distribution of epitopes within these motifs highlights their immunodominant nature and functional relevance. Additional conserved epitopes were also identified outside these motifs, including residues 80–115 and 142–153 in ectodomain A, 177–194 in ectodomain B, and 322–336 in ectodomain C.

However, linear predictions alone cannot fully capture the conformational and topological context of these epitopes within the native viral particle. In alphaviruses, epitope accessibility may be influenced by the quasi-3-fold (q3) symmetry unit. This structural organization integrates inter- and intradimer contacts that dictate whether predicted epitopes are surface-exposed or sterically occluded.^57^ To refine our prediction analysis and better approximate the native viral architecture, conserved epitopes were next mapped onto high-resolution q3 models of CHIKV and EEEV E2 proteins, assessing their spatial distribution and solvent accessibility.

### Structural Mapping of Conserved Epitopes onto the q3 Unit of Alphavirus E2 Proteins

To evaluate the structural accessibility of conserved epitopes, we mapped them onto the quasi-3-fold (q3) symmetry unit of the E2 glycoprotein, which represents the biologically relevant arrangement of E1–E2 heterodimers in the viral spike. The three-dimensional models used in this analysis exhibited high structural quality with satisfactory atomic organization and stereochemical validation (see methods/Supporting Information).

Several conserved epitopes were located within ectodomains A and B or to adjacent β-ribbons, regions typically solvent-exposed and frequently targeted by neutralizing antibodies.^24^ In CHIKV, multiple B-cell epitopes clustered at the apex and lateral surfaces of the q3 spike, supporting their potential accessibility to antibody binding (Figure 4).

**Figure 4:**
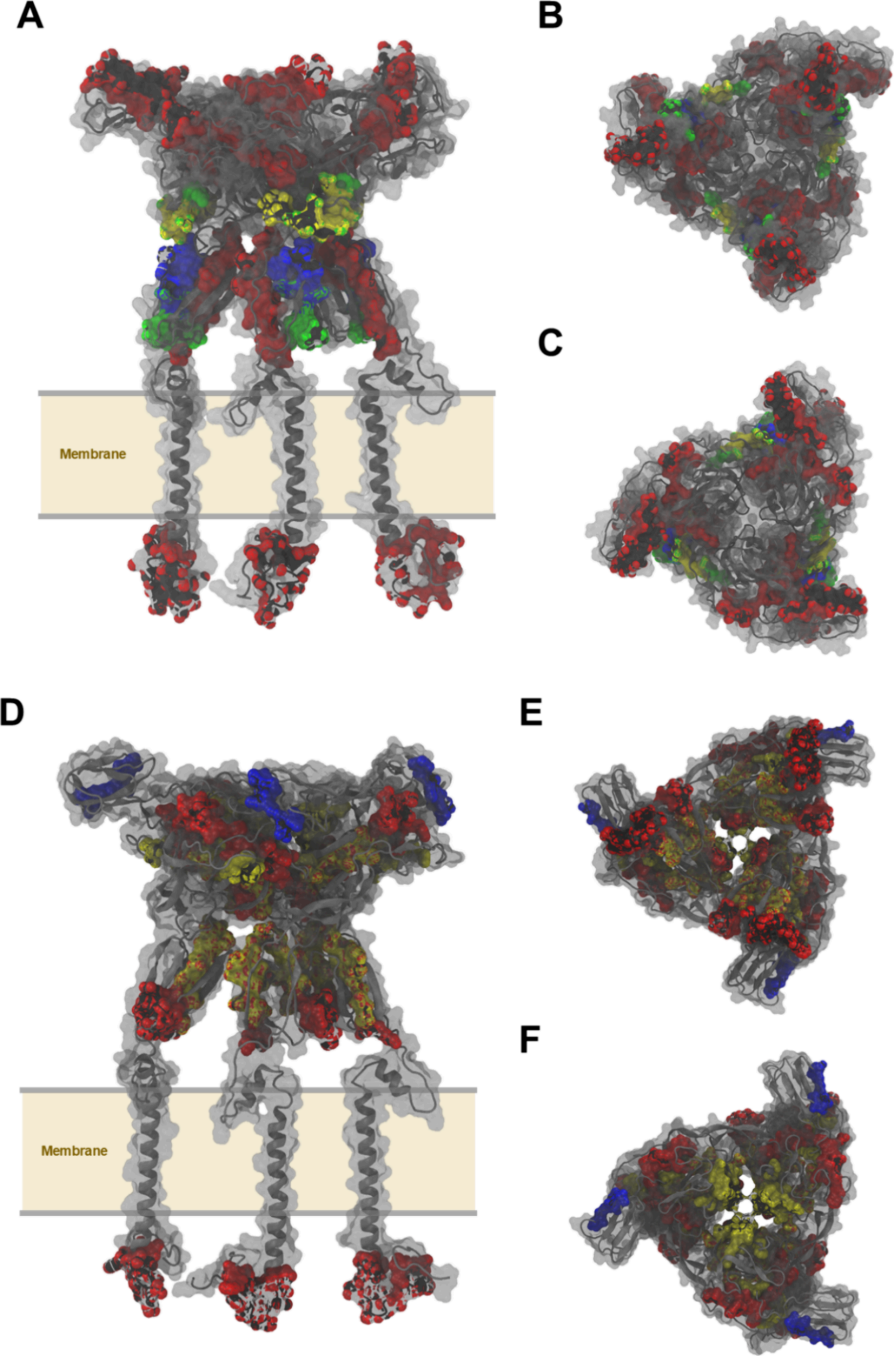
Three-dimensional mapping of predicted epitopes on the E2 glycoprotein of EEEV (A–C) and CHIKV (D–F). Structures were modeled using crystal data from the RCSB PDB (accessions 6MX4 and 6NK7, respectively). Predicted epitopes are highlighted as follows: B-cell linear epitopes (Bepipred, blue; PAP, yellow), Tc-cell epitopes (NetCTL, green), and Th-cell epitopes (MHCII Binding Predictions, red). Panels show longitudinal views of the E2 protein (A, D), top views rotated 90° over domains A and B (B, E), and lateral views rotated 90° over domain C (C, F).

The analysis of the surface accessibility (ASA) and buried surface area (BSA) of the mapped epitopes revealed important differences between the monomeric and quaternary (Q3) states (Table 2). In the CHIKV epitopes 80–92, 98–105, 109–115, and 282–297, high ASA values were considered in the monomer, ranging from 197.4 to 568.9 Å^2^, with a reduction in Q3 to values between 152.4 and 445.6 Å^2^. This reduction indicates that portions of these epitopes become occluded upon trimer formation, suggesting their involvement in interchain interfaces; this is also reinforced in Table S3 (Cocomaps and PISA in residue BSA analysis). The epitopes 282–297 stood out for the highest absolute ASA value in the monomer (568.9 ± 48.3 Å^2^), remaining relatively exposed in Q3 (445.6 ± 63.2 Å^2^).

**Table 2:** Relationship between the Total, Solvent-Accessible (ASA), and Buried Surface (BSA) Areas of Conserved B-Cell Epitopes Mapped onto the E2 Glycoproteins of CHIKV and EEEV^a^

organism	residues	sequence	**epitope area (Å** **^2^** **)**	**E2 monomer ASA (Å** **^2^** **)**	**q3 ASA (Å** **^2^** **)**	**q3 BSA (Å** **^2^** **)**
CHIKV	80–92	RAGLFVRTSAPCT	1737.92 ± 24.93	760.65 ± 26.57	705.40 ± 23.45	55.24 ± 3.57
CHIKV	98–105	GHFILARC	1359.75 ± 23.65	197.43 ± 32.72	152.38 ± 37.23	45.05 ± 5.07
CHIKV	109–115	ETLTVGF	1143.73 ± 6.65	256.27 ± 17.40	191.50 ± 21.53	64.77 ± 24.47
CHIKV	207–212	NEGLIT	984.13 ± 16.29	373.49 ± 31.14	372.84 ± 32.25	0.65 ± 1.13
CHIKV	237–244	YNSPLVPR	1307.29 ± 25.73	448.82 ± 21.03	146.94 ± 13.99	301.88 ± 15.10
CHIKV	282–297	QVIMLLYPDHPTLLSY	2302.06 ± 41.79	568.94 ± 48.29	445.58 ± 63.19	123.36 ± 31.28
EEEV	142–152	PEHGVELPCNR	1680.43 ± 0.99	689.29 ± 1.09	344.82 ± 11.74	344.47 ± 11.91
EEEV	323–332	LEYTWGNHPP	1396.89 ± 2.39	245.67 ± 1.42	222.58 ± 0.77	23.09 ± 2.09

a The total surface area and ASA were calculated for the isolated epitopes and the monomeric E2 protein, respectively. Within the context of the q3 unit, both the ASA and BSA of each epitope are reported. The standard deviation corresponds to the values obtained across the three chains of the E2 trimer.

The 207–212 epitopes maintained practically unchanged ASA between the monomer (373.5 ± 31.1 Å^2^) and Q3 (372.8 ± 32.3 Å^2^), with low BSA (0.65 ± 1.13 Å^2^). In contrast, the 237–244 epitope showed a reduction from 448.8 ± 21.0 Å^2^ in the monomer to 146.9 ± 13.9 Å^2^ in Q3, accompanied by high BSA. In the EEEV epitopes, segment 142–152 showed high ASA in the monomer (689.3 ± 1.1 Å^2^), with a decrease in Q3 (344.8 ± 11.7 Å^2^) and high BSA (344.5 ± 11.9 Å^2^). Meanwhile, epitope 323–332 showed interesting ASA in the monomer (245.7 ± 1.4 Å^2^) and Q3 (222.6 ± 0.8 Å^2^), with relatively low BSA (23.1 ± 2.1 Å^2^). The principle that the quaternary organization of the E2 glycoprotein modulates epitope exposure, rendering certain regions highly accessible while burying others, is strongly corroborated by a study of a chikungunya virus-like particle (VLP) vaccine, which enhances our understanding of how the native virion architecture dictates epitope availability and immunogenicity.^24^

The combined evaluation of ASA and BSA provides a detailed view of the structural accessibility of epitopes for antibody interaction (see the detailed characterization in the supplementary Excel file). A high-resolution cryo-EM study of broadly neutralizing monoclonal antibodies, such as 506.A08 and 506.C01, revealed that these antibodies target the highly exposed apex of the E2 glycoprotein B domain.^24^ Interestingly, they engage this same accessible region from distinct angles, defining unique neutralizing sites that differ from those of previously characterized antibodies targeting the lateral tip of the B domain. This finding underscores the structural versatility and cross-binding potential of neutralizing antibodies toward exposed epitopes.^24,58^

The identification of B-cell epitopes with consistent solvent exposure strengthens the reliability of sequence-based predictions by anchoring them to the structural context of the virion. This information is particularly relevant for vaccine design and diagnostic development targeting alphavirus E2 glycoproteins,^59^ as neutralizing antibodies are the primary correlation of protection against CHIKV infection.^60^

However, epitope recognition and antibody binding depend not only on solvent exposure and spatial arrangement but also on local energetic interactions.^22,23^ To explore this, we next analyzed hydrogen-bonding patterns within conserved epitopes to determine how donor–acceptor networks contribute to structural stability and antibody binding on the E2 glycoprotein.

### Hydrogen Bond Donors and Acceptors on Conserved Epitope Surfaces

Recent high-definition structural characterizations underscore the relevance of features of neutralizing antibody–CHIKV epitope interfaces.^24^ A simplified view of the interface is presented in the Supporting Information (Figure S3), providing a limited structural snapshot of the interaction based on exposed donor and acceptor features.

The interface analysis was conducted using the comprehensive interaction (Figure S3), which is designed to identify a full spectrum of noncovalent contacts, including conventional and carbon–hydrogen bonds, halogen bonds, electrostatic interactions (salt bridges and repulsive charges), hydrophobic interactions (alkyl and π–alkyl), π interactions (π–π stacked/T-shaped and π–cation), and van der Waals forces. Despite this broad analytical capacity, our analysis of the neutralizing antibody–epitope interface (PDB ID: 8dww) revealed that its stability is specifically governed by a limited set of five key interactions: two conventional hydrogen bonds, two carbon–hydrogen bonds, and one complementary polar charge interaction. This finding underscores the highly specific physicochemical nature of this particular complex, highlighting that its stabilization relies on a precise network of donor/acceptor-dependent contacts rather than broader hydrophobic or π-stacking forces. Consequently, the emphasis placed on the hydrogen bond donors and acceptors in Figure 5 is a direct reflection of their critical and predominant roles in defining the stability of this specific neutralizing interface.

**Figure 5:**
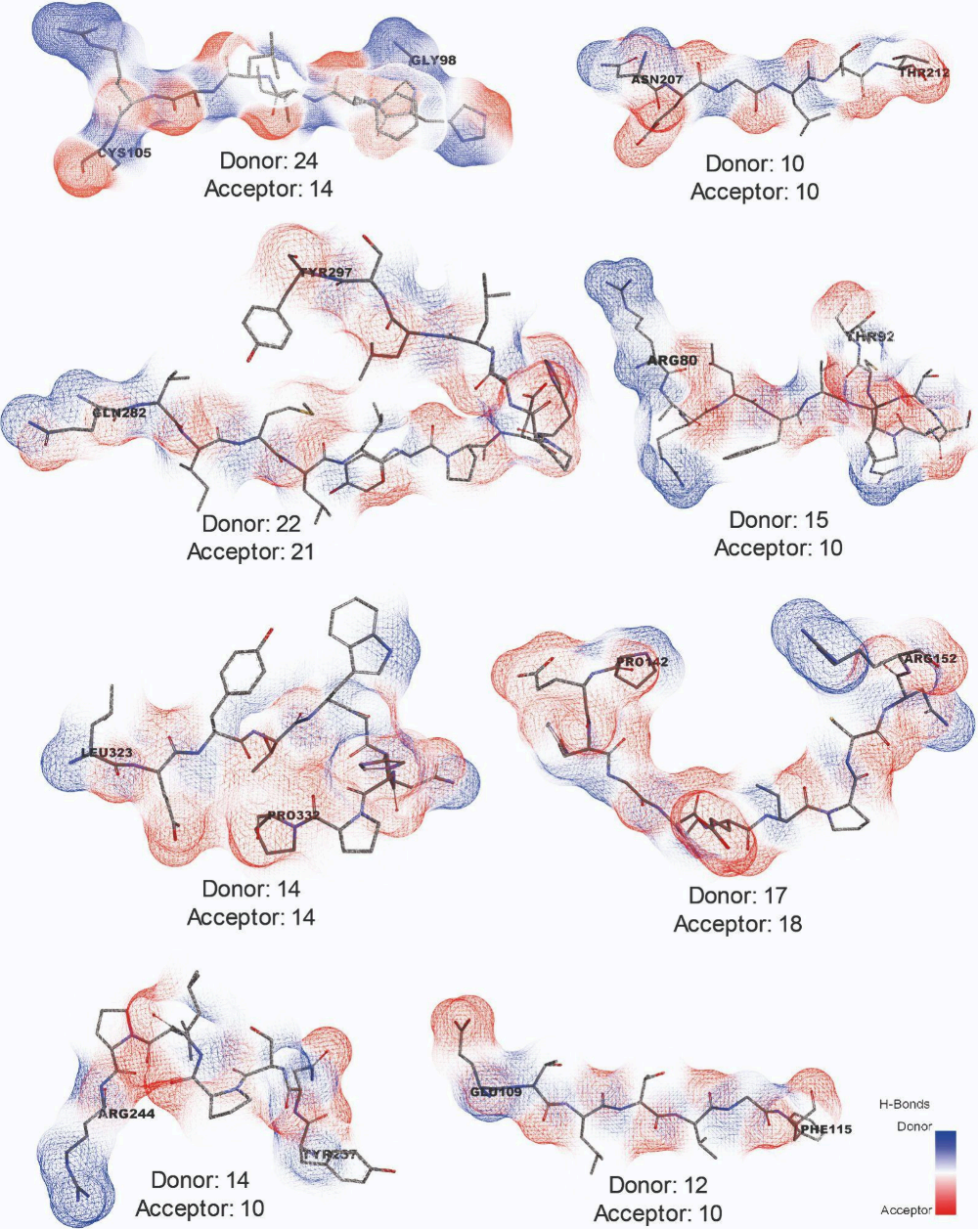
Mapping of potential hydrogen donors and acceptors in accessible alphavirus B-cell epitopes. Surface mesh representation of the spatial distribution of hydrogen acceptors (red) and donors (blue) with an intensity gradient (white = neutral). Stick amino acid residues represent each viral epitope. N- and C-terminal portions carry the amino acid number and residue identifier. The higher resolution of the structural mesh highlights key residues, e.g., lysine and glutamic acid.

Among the eight surface-exposed B-cell epitopes, hydrogen bond donors (128) exceeded acceptors (107) (Figure 5). This imbalance was particularly evident in epitopes such as CHIKV 98–105 (24:14), which may favor interactions with antibody complementarity-determining regions (CDRs) enriched in residues such as tyrosine or serine. The ability to form hydrogen bonds is crucial for molecular stability, solubility, receptor binding, and membrane permeability.^61,62^ In contrast, epitopes with balanced donor/acceptor ratios, such as EEEV 323–332 (14:14), may support bidirectional hydrogen bonding, potentially enhancing antibody avidity.

Mapping these hydrogen-bonding patterns within the q3 unit highlighted structural asymmetries that influence the immunogenic potential. Hydrogen bond density varied across epitopes and domains: epitopes in ectodomain A showed higher stabilization, whereas those in β-ribbons and ectodomain C exhibited fewer polar contacts and greater conformational flexibility.

## Discussion

Structural similarities across the E2 glycoproteins of CHIKV and EEEV underscore their evolutionary conservation and functional relevance in Alphavirus biology.^63,64^ Such conserved features can be exploited for the rational design of cross-protective vaccines by prioritizing epitopes that are both structurally and antigenically stable.^65–67^ In this study, we identified 39 conserved B- and T-cell epitopes within the E2 ectodomains, delineating critical regions that may contribute to viral neutralization and broad-spectrum immune responses. Conserved targets represent promising putative candidates for therapeutic intervention^64^ and may facilitate the development of vaccines capable of inducing cross-protective immunity against phylogenetically related arboviruses.^2,68^

The conserved B-cell and T-cell epitopes identified in this study were distributed across all three ectodomains, including both the N- and C-terminal regions. This observation is consistent with previous reports, which demonstrate the presence of immunogenic peptides in the terminal regions of the E2 protein.^69^ Notably, in the C ectodomain of E2, we predicted several CD4^+^ T helper epitopes, including one that overlapped with a B-cell epitope, suggesting a region of integrated humoral and cellular immune recognition. Consistently, experimental studies have shown that the full-length CHIKV E2 protein elicits stronger immune responses in mice and may enhance the efficacy of subunit vaccines compared to truncated variants that lack the C-terminal epitopes.^69^

Among the identified epitopes, the majority were predicted to bind MHCII molecules and activate CD4^+^ T helper responses with nearly half overlapping conserved antigenic motifs. Additionally, two EEEV epitopes were predicted to elicit CD8^+^ cytotoxic T-cell responses. Because the T-cell epitope content contributes substantially to overall antigenicity,^69^ these responses would complement neutralizing antibody activity, thereby enhancing both the breadth and durability of protective immunity.^70^

Neutralizing antibody induction remains the cornerstone of effective alphavirus vaccines, as antibody titers strongly correlate with protection against infection.^70^ While cross-neutralizing E2-specific monoclonal antibodies (mAbs) have been reported for arthritogenic alphaviruses,^71^ comparable mAbs have not yet been identified for encephalitic alphaviruses.^13^ Moreover, defining conserved antibody targets shared across both virus groups remains a major challenge in the field.

In this context, we identified eight conserved and surface-exposed B-cell epitopes across the E2 glycoproteins of CHIKV and EEEV. Most putative CHIKV epitopes clustered within ectodomains A and B or in the connecting β-ribbons, structural regions that are solvent-exposed and frequently recognized by neutralizing antibodies.^72^ Ectodomain A, in particular, is highly accessible, interacts with antibody Fab regions,^50^ and mediates both receptor engagement and viral entry.^73^ Ectodomain B, in turn, contributes to the structural integrity of the E1–E2 spikes^73^ and harbors conserved residues recognized by neutralizing antibodies. Despite its potential as an important target for immunogen design, no conserved B epitopes were identified for the B ectodomain of EEEV. This observation is consistent with prior studies reporting that the A and B ectodomains of E2 elicit antibodies in pan-arthritogenic alphaviruses but not in encephalitogenic alphaviruses, suggesting that one cause is a disparity in the sequence similarity of the A and B domains of the E2 proteins, which is lower in encephalitic than in arthritogenic alphaviruses.^74^

Structural mapping in the quaternary context further emphasized the need to evaluate epitopes within their native organization.^75–77^ Cryo-EM studies of Mayaro virus at 4.4 Å resolution revealed that the spike quasi-3-fold (q3) unit—formed by trimers of E1–E2 heterodimers arranged along icosahedral symmetry axes—provides the relevant framework for epitope exposure and accessibility.^16^ More recently, Bandyopadhyay et al. identified in a mouse model a human IgG1 that requires bivalency to recognize a quaternary epitope on the E2 glycoprotein, bridging spikes across the icosahedral 2-fold axis through a unique binding mode.^78^ Consistently, in our analysis, epitopes accessible in isolated E2 chains became partially buried within the q3 assembly. However, since our analyses were based on a single static conformational model, they may not fully capture the intrinsic dynamics of alphavirus glycoproteins, which undergo structural rearrangements during viral maturation and host cell entry. Therefore, molecular dynamics simulations could provide complementary insights into whether these conserved epitopes remain solvent-accessible across different conformational states.

Beyond solvent exposure, the physicochemical environment of epitopes also influences their structural stability and immunogenic potential.^79^ Hydrogen bond profiling in our study revealed that conserved epitopes form extensive donor–acceptor networks. Some epitopes displayed pronounced asymmetry (e.g., CHIKV 98–105), favoring directional interactions with antibody CDRs enriched in polar residues. In contrast, other epitopes (e.g., EEEV 323–332) exhibited more balanced donor–acceptor distributions, predicted to favor bidirectional hydrogen bonding and potentially enhancing avidity.^61,80^ High densities of hydrogen donors and acceptors in epitopes CHIKV 282–297 and EEEV 142–152 align with experimentally validated neutralizing antibody-binding sites, exemplified by the CHIKV–Fab complex (PDB: 7CW2). These findings suggest that the hydrogen-bonding capacity contributes not only to the local stabilization of epitopes but also to their ability to engage antibodies effectively.

The conserved epitopes identified in this study provide a rational foundation for the design of chimeric, multiepitope vaccine constructs optimized according to the native quaternary architecture of alphavirus E2. When combined with molecular linkers and adjuvants, such constructs could enhance immunogenicity by simultaneously activating innate and adaptive immune responses, a critical requirement for effective vaccine development.^81^ To maximize their translational potential, these candidates should undergo further *in silico* refinement through complementary analyses, including multiepitope vaccine sequence design, molecular modeling, and advanced physicochemical characterization, molecular docking and molecular dynamics of protein–protein interaction with host immune receptors, population coverage analysis, immune simulations, codon optimization, and virtual cloning of the final vaccine construct.^82,83^ This integrative computational framework, when complemented by experimental validation in a wet lab, represents a powerful strategy that is reshaping modern vaccine development.^84^

Finally, we introduce the POA, a semiautomated computational framework designed to integrate heterogeneous outputs from multiple immunoinformatics tools into a unified and reproducible data set (10.5281/zenodo.15330709). This pipeline streamlines the epitope prediction process by reducing manual curation requirements, improving accessibility for researchers with limited computational expertise, and accelerating the identification of high-potential antigenic targets. The POA enables large-scale screening of viral protein epitopes by systematically consolidating results from B- and T-cell epitope predictors, as well as conservation analysis tools through its POA1 and POA2 modules. Beyond alphaviruses, this framework represents a transferable strategy for rational vaccine design against a broad spectrum of emerging and re-emerging pathogens, including flaviviruses and coronaviruses. Future developments aim to expand POA functionality by incorporating additional predictive layers and integrating these data sets into comprehensive *in silico* platforms for vaccine and diagnostic development.

Importantly, our innovative use of the q3 unit for structural mapping illustrates the synergy between sequence-based predictions and structural validation, enabled by recent advances in cryo-EM. This integrative approach enhances confidence in epitope accessibility and antigenicity, ultimately improving the translational value of in silico predictions for vaccine development.

## Limitations of the Study

As a limitation of this study, the epitope mapping relied on the currently available crystallographic and cryo-EM structures of CHIKV and EEEV E2 proteins, which have resolution constraints and may not fully capture the most accurate conformations of the protein. Consequently, the spatial positioning and accessibility of epitopes might be affected by these structural limitations. The future availability of higher-resolution structures could enable a re-evaluation of our findings and enhance the reliability of the three-dimensional insights. Also, future work could build upon our findings by performing a detailed sensitivity analysis of the geometric cutoffs used for the hydrogen bond definition, potentially revealing the contributions of weaker or more transient hydrogen bonds to complex stability. Furthermore, although multiple complementary approaches support our computational predictions, they remain theoretical and require experimental validation in relevant biological systems to confirm their immunogenicity and functional relevance.

## Conclusions

This study systematically identified 39 conserved linear B- and T-cell epitopes within the E2 glycoproteins of CHIKV and EEEV and structurally validated them using an integrated immunoinformatics pipeline. The distribution of these epitopes across all ectodomains of E2 highlights immunogenic regions with the potential to elicit broad and durable immune responses. These epitopes represent promising candidates for the development of multiepitope vaccine strategies aimed at cross-protective immunity against alphaviruses of medical and veterinary relevance.

By incorporating quaternary structural mapping through the quasi-3-fold unit, we demonstrate that epitope accessibility is determined not only by sequence conservation but also by the native architecture of the viral spike. This integrative approach strengthens confidence in computational predictions and provides a rational framework for prioritizing epitopes with the highest translational potential. Beyond alphaviruses, the methodology established here provides a transferable platform to accelerate the rational design of vaccines against other emerging viral pathogens.

## Supplementary Material





## Data Availability

Detailed outputs from epitope prediction and screening using the POA1 and POA2 modules are available at https://github.com/UbiratanBatista/POA_Project. Supplementary methods and computational workflows are available in the Zenodo repository to ensure full reproducibility and transparency of all analyses (10.5281/zenodo.14968128).

## Notes

Reference sequences of E2 glycoproteins (Box S1); list of predicted T helper epitopes restricted to multiple HLA alleles (Table S1), as well as comprehensive, nonredundant list of predicted B-, Tc-, and Th-cell epitopes for CHIKV and EEEV following conservancy analyses and membrane topology predictions (Table S2); structural validation of E2 crystal models from CHIKV and EEEV (Figures S1 and S2); (Figure S3) representative example of the binding interface between a Fab antibody and E2 epitopes; calculations of the solvent-accessible surface area (SASA) and buried surface area (BSA) for epitopes located at the E1–E2 dimer interfaces performed using both PDBePISA and COCOMAPS, ensuring cross-validation of molecular interface analyses (Table S3) (PDF)
Residue-level SASA and BSA data for each epitope (XLSX)

## δ

Renewable Carbon and Biological Systems (ReCABS) Laboratory, Department of Biotechnology, Lorena School of Engineering, University of São Paulo (EEL-USP), 12602-810 Lorena, SP, Brazil
