# Supplementary material for: In Silico Characterization of Conserved Epitopes in Alphavirus E2 Proteins: A Promising Approach for Pan-vaccine Design
Source: ACS Omega. 2025 Nov 26;10(48):58856–68. doi: 10.1021/acsomega.5c07474 (PMC12771413; doi:10.1021/acsomega.5c07474)
Supplement: Supplementary file 1 [file ao5c07474_si_001.pdf]

Supporting Information

In silico characterization of conserved epitopes in Alphavirus E2 proteins: A promising approach for pan-vaccine design

Ubiratan da Silva Batista [a δ], Ana Clara Gomes de Souza[a], Breno de Mello Silva[a]*, and Ricardo Lemes Gonçalves[a]*

1. *Laboratory of Biology and Technology of Microorganisms (LBTM), Department of Biological Sciences, Federal University of Ouro Preto (UFOP), 35400-000 Ouro Preto, MG, Brazil*

* To whom correspondence should be addressed:

breno@ufop.edu.br and ricardolemesg@gmail.com.

Present address:

[δ]U.S.B.: Renewable Carbon and Biological Systems (ReCABS) Laboratory, Department of Biotechnology, Lorena School of Engineering, University of São Paulo (EEL-USP), 12602-810 Lorena, SP, Brazil

Box S1. Amino acid sequences of the E2 glycoproteins from CHIKV and EEEV. Sequences are provided in FASTA format and correspond to the full-length E2 proteins used for epitope prediction and structural analysis in this study. These reference sequences were obtained from the NCBI database under accession numbers NP_690589.2 (CHIKV) and NP_632022.1 (EEEV).

**Sequence in FASTA format**
>E2_PROTEIN_CHIKV_NP_690589.2<br>STKDNFNVYKATRPYLAHCPDCGEGHSCHSPVALERIRNEATDGTLKIQVSLQIGIGTDDSHDWTKLRY<br>MDNHIPADAGRAGLFVRTSAPCTITGTMGHFILARCPKGETLTVGFTDSRKISHSCTHPFHHDPPVIGREK<br>FHSRPQHGKELPCSTYVQSNAATAEEIEVHMPPDTPDRTLLSQQSGNVKITVNSQTVRYKCNCGGSNEG<br>LITTDKVINNCKVDQCHAAVTNHKKWQYNSPLVPRNAELGDRKGKIHIPFPLANVTCMVPKARNPTVT<br>YGKNQVIMLLYPDHPTLLSYRSMGEEPNYQEEWVTHKKEVVLTVPTEGLEVTWGNNEPYKYWPQLSA<br>NGTAHGHPHEIILYYYELYPTMTVVVVSVASFILLSMVGMAVGMCMCARRRCITPYELTPGATVPFLLS<br>LICCIRTAKA
>E2_PROTEIN_EEEV_NP_632022.1<br>DLDTHFTQYKLARPYIADCPNCGHSRCDSPIAIEEVRGDAHAGVIRIQTSAMFGLKTDGVDLAYMSFMN<br>GKTQKSIKIDNLHVRTSAPCSLVSHHGYYILAQCPPGDTVTVGFHDGPNRHTCTVAHKVEFRPVGREKY<br>RHPPEHGVELPCNRYTHKRADQGHYVEMHQPGLVADHSLLSIHSAKVKITVPSGAQVKYYCKCPDVRE<br>GTTSSDYTTTCTDVKQCRAYLIDNKKWVYNSGRLPRGEGDTFKGKLHVPFVPVKAKCIATLAPEPLVEH<br>KHRTLILHLYPDHPTLLTTRSLGSDANPTRQWIERPTTVNFTVTGEGLEYTWGNHPPKRVWAQESGEGN<br>PHGWPHEVVVYYYNRYPLTTIIGLCTCVAIIMVSCVTSVWLLCRTRNLCITPYKLAPNAQVPILLALLCCI<br>KPTRA

Table S1. Conserved $CD4^+$ T helper (Th) cell epitopes from the E2 glycoproteins predicted to bind multiple HLA-DR alleles. Epitopes (with identity $\geq$ 60%) were identified using the IEDB MHCII-Binding Predictions tool and retained when they demonstrated binding affinity to two or more HLA-DR alleles. Start and end positions correspond to amino acid numbering in the reference E2 protein sequences of CHIKV (NP_690589.2) and EEEV (NP_632022.1).

Species	Start position	End position	Sequence*	HLA allele
CHIKV	6	20	FNVYKATRPYLAHCP	HLA-DRB1*01:01
				HLA-DRB1*07:01
	98	112	GHFILARCPKGETLT	HLA-DRB1*01:01
				HLA-DRB1*11:01
	99	113	HFILARCPKGETLTV	HLA-DRB1*01:01
				HLA-DRB1*11:01
	281	295	NQVIMLLYPDHPTLL	HLA-DRB1*15:01
				HLA-DRB4*01:01
	282	296	QVIMLLYPDHPTLLS	HLA-DRB1*15:01
				HLA-DRB4*01:01
EEEV	6	20	FTQYKLARPYIADCP	HLA-DRB1*01:01
				HLA-DRB1*07:01
				HLA-DRB5*01:01
	174	188	DHSLLSIHSAKVKIT	HLA-DRB1*01:01
				HLA-DRB1*07:01
				HLA-DRB1*13:02
				HLA-DRB5*01:01
	175	189	HSLLSIHSAKVKITV	HLA-DRB1*01:01
				HLA-DRB1*07:01
				HLA-DRB1*13:02
				HLA-DRB5*01:01
	176	190	SLLSIHSAKVKITVP	HLA-DRB1*01:01
				HLA-DRB1*07:01
				HLA-DRB5*01:01
	177	191	LLSIHSAKVKITVPS	HLA-DRB1*01:01
				HLA-DRB1*07:01
	393	407	CITPYKLAPNAQVPI	HLA-DRB1*01:01
				HLA-DRB1*13:02
	394	408	ITPYKLAPNAQVPIL	HLA-DRB1*01:01
				HLA-DRB1*09:01
				HLA-DRB1*13:02

	395	409	TPYKLAPNAQVPILL	HLA-DRB1*01:01
				HLA-DRB1*09:01
				HLA-DRB1*13:02
	396	410	PYKLAPNAQVPILLA	HLA-DRB1*01:01
				HLA-DRB1*13:02
	397	411	YKLAPNAQVPILLAL	HLA-DRB1*01:01
				HLA-DRB1*13:02

Table S2. Conserved epitopes within the E2 glycoproteins of CHIKV and EEEV identified using the POA pipeline. Epitopes were predicted with multiple tools (Bepipred, PAP-IMED, NetCTL, and IEDB MHCII-Binding Predictions) and further analyzed for sequence conservation (≥60% identity) and topological location based on transmembrane region prediction. Start and end positions refer to amino acid numbering in the reference E2 sequences of CHIKV (NP_690589.2) and EEEV (NP_632022.1). External and internal topology indicate whether the predicted epitope is likely exposed to solvent or embedded in the transmembrane region.

Species	Prediction method	Start position	End position	Sequence	Sequence length	Sequence identity (%)	External topology	Internal topology
CHIKV	Bepipred	207	212	NEGLIT	6	66,67	x	
CHIKV	PAP	80	92	RAGLFVRTSAPCT	13	61,54	x	
CHIKV	PAP	98	105	GHFILARC	8	62,5	x	
CHIKV	PAP	109	115	ETLTVGF	7	71,43	x	
CHIKV	PAP	237	244	YNSPLVPR	8	62,5	x	
CHIKV	PAP	282	297	QVIMLLYPDHPTLLSY	16	62,5	x	
CHIKV	PAP	404	419	PGATVPFLLSLICCIR	16	62,5		x
CHIKV	MHCII-BP	6	20	FNVYKATRPYLAHCP	15	60	x	
CHIKV	MHCII-BP	8	22	VYKATRPYLAHCPDC	15	60	x	
CHIKV	MHCII-BP	98	112	GHFILARCPKGETLT	15	60	x	
CHIKV	MHCII-BP	99	113	HFILARCPKGETLTV	15	60	x	
CHIKV	MHCII-BP	280	294	KNQVIMLLYPDHPTL	15	60	x	
CHIKV	MHCII-BP	281	295	NQVIMLLYPDHPTLL	15	66,67	x	

CHIKV	MHCII-BP	282	296	QVIMLLYPDHPTLLS	15	66,67	x	
CHIKV	MHCII-BP	283	297	VIMLLYPDHPTLLSY	15	66,67	x	
CHIKV	MHCII-BP	289	303	PDHPTLLSYRSMGEE	15	66,67	x	
CHIKV	MHCII-BP	290	304	DHPTLLSYRSMGEEP	15	60	x	
CHIKV	MHCII-BP	291	305	HPTLLSYRSMGEEPN	15	60	x	
CHIKV	MHCII-BP	397	411	ITPYELTPGATVPFL	15	66,67		x
CHIKV	MHCII-BP	398	412	TPYELTPGATVPFLL	15	66,67		x
CHIKV	MHCII-BP	399	413	PYELTPGATVPFLLS	15	60		x
EEEV	Bepipred	323	332	LEYTWGNHPP	10	70	x	
EEEV	PAP	142	152	PEHGVELPCNR	11	63,64	x	
EEEV	NetCTL	145	153	GVELPCNRY	9	66,67	x	
EEEV	NetCTL	317	325	TVTGEGLEY	9	66,67	x	
EEEV	MHCII-BP	6	20	FTQYKLARPYIADCP	15	60	x	
EEEV	MHCII-BP	8	22	QYKLARPYIADCPNC	15	60	x	
EEEV	MHCII-BP	9	23	YKLARPYIADCPNCG	15	66,67	x	
EEEV	MHCII-BP	174	188	DHSLLSIHSAKVKIT	15	60	x	
EEEV	MHCII-BP	175	189	HSLLSIHSAKVKITV	15	60	x	
EEEV	MHCII-BP	176	190	SLLSIHSAKVKITVP	15	60	x	
EEEV	MHCII-BP	177	191	LLSIHSAKVKITVPS	15	66,67	x	
EEEV	MHCII-BP	277	291	HRTLILHLYPDHPTL	15	60	x	
EEEV	MHCII-BP	278	292	RTLILHLYPDHPTLL	15	66,67	x	
EEEV	MHCII-BP	392	406	LCITPYKLAPNAQVP	15	66,67		x
EEEV	MHCII-BP	393	407	CITPYKLAPNAQVPI	15	66,67		x
EEEV	MHCII-BP	394	408	ITPYKLAPNAQVPIL	15	66,67		x
EEEV	MHCII-BP	395	409	TPYKLAPNAQVPILL	15	66,67		x
EEEV	MHCII-BP	396	410	PYKLAPNAQVPILLA	15	60		x
EEEV	MHCII-BP	397	411	YKLAPNAQVPILLAL	15	60		x

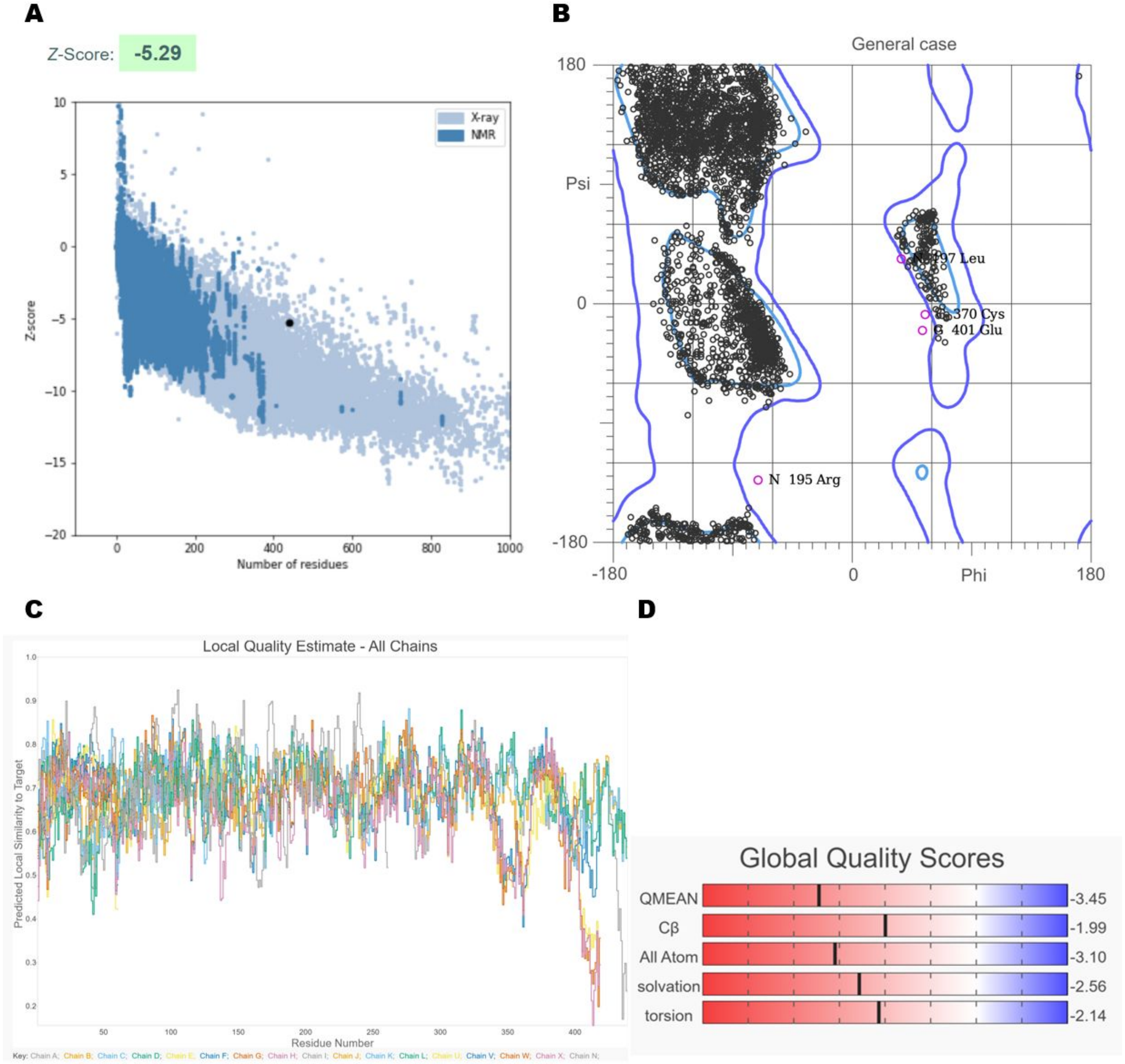

Figure S1. Quality assessment of the CHIKV E2 quaternary structure within the q3 unit (PDB ID: 6NK7, 4.99 Å). (A) Global model quality evaluated by ProSA-web Z-score. (B) Ramachandran plot generated with MolProbity showing protein backbone stereochemistry. (C) Local quality profile and (D) global quality scores obtained from QMEAN analysis, performed on the original PDB file after removal of ligand groups. Together, these complementary assessments support the reliability of the CHIKV E2 model for structural analysis and epitope mapping.

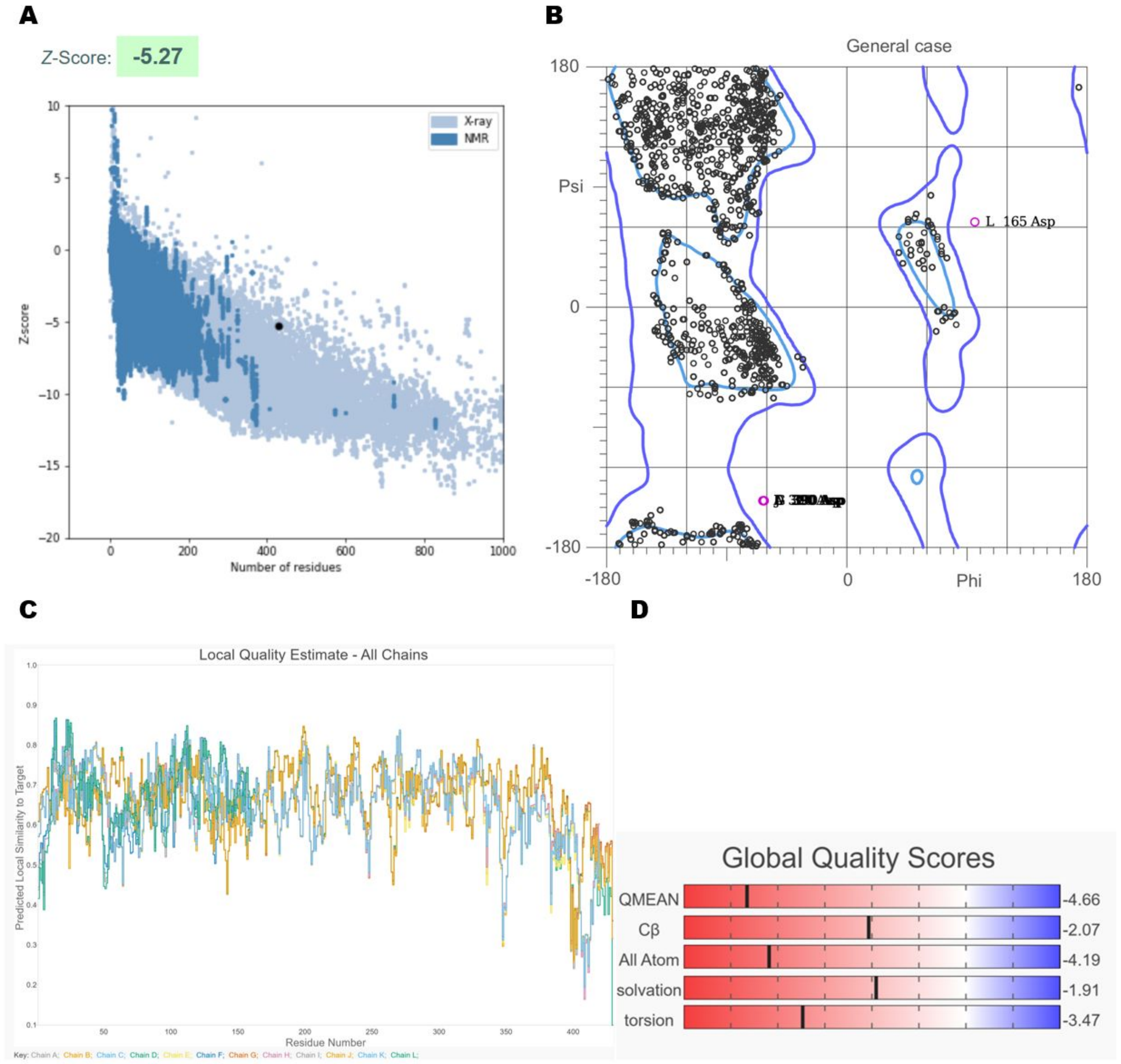

Figure S2. Quality assessment of the EEEV E2 quaternary structure within the q3 unit (PDB ID: 6MX4, 4.40 Å). (A) Global model quality evaluated by ProSA-web Z-score. (B) Ramachandran plot generated with MolProbity showing protein backbone stereochemistry. (C) Local quality profile and (D) global quality scores obtained from QMEAN analysis, performed on the original PDB file after removal of ligand groups. Together, these complementary assessments support the reliability of the EEEV E2 model for structural analysis and epitope mapping.

Table S3. Comparative analysis of intratrimeric chain interfaces of the E2 glycoprotein epitopes using CoCoMaps2.0 and PDBe-PISA. The table reports the calculated complex accessible surface area (ASA), free ASA, buried ASA, and percentage of buried ASA for selected residues within epitopes located at chain–chain interfaces (E–F, E–G, and F–G). Values are shown for both platforms to illustrate their consistency. (see detailed characterization in the supplementary Excel file: https://docs.google.com/spreadsheets/d/1hCqxQbyKG6ec5oIghwCePu4f-k6ZJIhC/edit?usp=drive_link&ouid=104337351218678400469&rtpof=true&sd=true)

Res. Name	Res. Number	Epitope	Interface	Chain	Cocomaps 2.0				PDBe-PISA			
					Complex ASA	Free ASA	Buried ASA (Interface)	Buried ASA %	Complex ASA	Free ASA	Buried ASA (Interface)	Buried ASA %
ALA	89	RAGLFVRTSAPCT	E vs F	E	14,9	29,09	14,19	48,8	15,15	29,88	14,73	49,30
PRO	90	RAGLFVRTSAPCT	E vs F	E	91,21	101,41	10,2	10,1	91,2	101,08	9,88	9,77
ARG	104	GHFILARC	E vs F	E	73,05	123,45	50,4	40,8	73,18	124,01	50,83	40,99
ASP	290	QVIMLLYPDHPTLLSY	E vs F	E	98,15	115,99	17,84	15,4	98,26	116,23	17,97	15,46
SER	88	RAGLFVRTSAPCT	E vs F	F	66,71	93,27	26,56	28,5	66,8	93	26,2	28,17
GLU	109	ETLTVGF	E vs F	F	61,18	107,71	46,53	43,2	61,27	106,63	45,36	42,54
ARG	86	RAGLFVRTSAPCT	E vs G	E	74,8	101,13	26,33	26	73,97	100,24	26,27	26,21
GLU	109	ETLTVGF	E vs G	E	59,38	121,86	62,48	51,3	58,42	120,43	62,01	51,49
THR	110	ETLTVGF	E vs G	E	23,13	53,82	30,69	57	22,91	53,55	30,64	57,22
ALA	89	RAGLFVRTSAPCT	E vs G	G	24,07	44,85	20,78	46,3	24,17	45,26	21,09	46,60
PRO	90	RAGLFVRTSAPCT	E vs G	G	71,47	83,4	11,93	14,3	70,15	82,87	12,72	15,35
ARG	104	GHFILARC	E vs G	G	107,89	148,98	41,09	27,6	108,93	150,29	41,36	27,52
ASP	290	QVIMLLYPDHPTLLSY	E vs G	G	62,55	96,31	33,76	35,1	62,75	97,48	34,73	35,63
HIS	291	QVIMLLYPDHPTLLSY	E vs G	G	10,69	24,53	13,84	56,4	10,65	24,02	13,37	55,66
PRO	90	RAGLFVRTSAPCT	F vs G	F	68,07	89,45	21,38	23,9	67,29	88,7	21,41	24,14
ARG	104	GHFILARC	F vs G	F	96,85	139,53	42,68	30,6	94,62	137,57	42,95	31,22
ARG	86	RAGLFVRTSAPCT	F vs G	G	79,32	98,76	19,44	19,7	80,11	99,79	19,68	19,72

GLU	109	ETLTVGF	F vs G	G	81,6	118,56	36,96	31,2	81,13	117,22	36,09	30,79
THR	110	ETLTVGF	F vs G	G	36,92	47,53	10,61	22,3	37,49	48,25	10,76	22,30

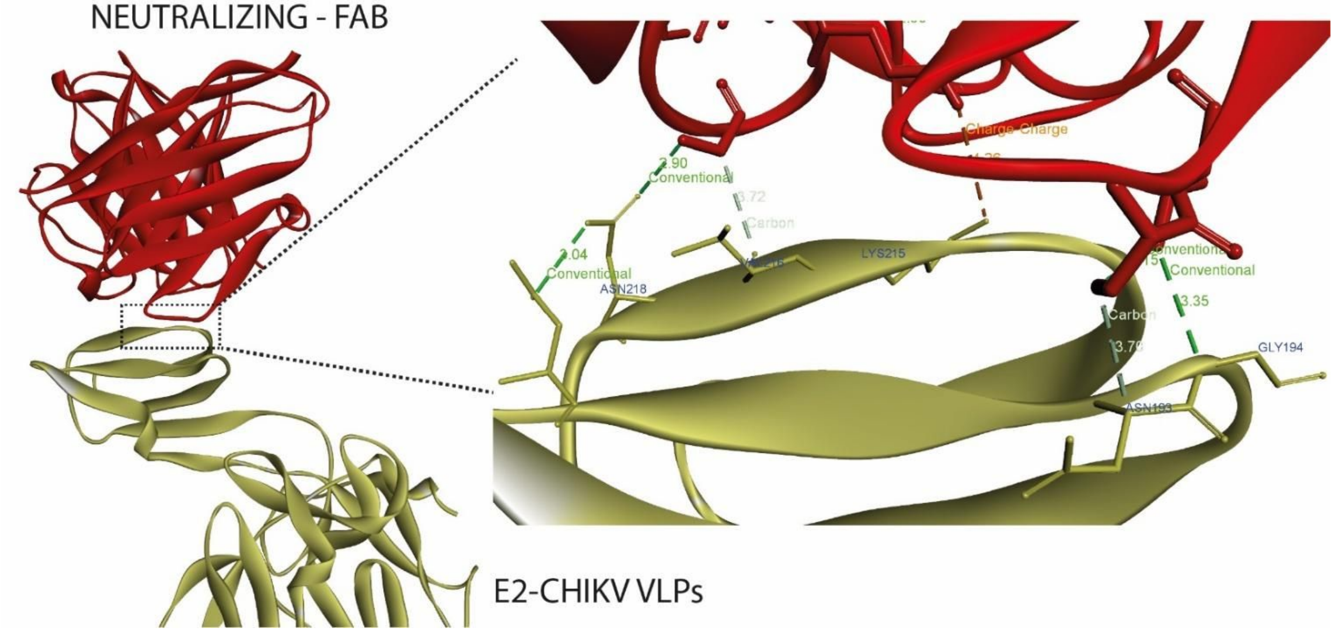

Figure S3. Neutralizing antibody-epitope binding interface. The crystal PDB ID: 8dww reveals that binding is stabilized by a network of five key donor/acceptor-dependent interactions: two conventional hydrogen bonds, two carbon-hydrogen bonds, and one complementary polar charge interaction, illustrating the critical role of well-positioned physicochemical bonds in stabilizing the antibody-antigen complex. The Fab portion of the antibody is represented in newcarton in red, and the E2 binding portion is represented in yellow. The respective residues involved in the interaction are represented in sticks.
